# Chinese version of the Perceived Stress Scale-10: A psychometric study in Chinese university students

**DOI:** 10.1371/journal.pone.0189543

**Published:** 2017-12-18

**Authors:** Wei Lu, Qian Bian, Wenzheng Wang, Xiaoling Wu, Zhen Wang, Min Zhao

**Affiliations:** 1 Shanghai Mental Health Center, Shanghai Jiao Tong University School of Medicine, Shanghai, P.R. China; 2 Academic Affairs Office, Shanghai Jiao Tong University, Shanghai, P.R. China; Xi′an Jiaotong University School of Medicine, CHINA

## Abstract

Chinese university students often suffer from acute stress, which can affect their mental health. We measured and evaluated perceived stress in this population using the Simplified Chinese version of the 10-item Perceived Stress Scale (SCPSS-10). The SCPSS-10, Patient Health Questionnaire (PHQ), and Generalized Anxiety Disorder 7-item scale (GAD-7) were conducted in 1096 university students. Two weeks later, 129 participants were re-tested using the SCPSS-10. Exploratory factor analysis yielded two factors with Eigen values of 4.76 and 1.48, accounting for 62.41% of the variance. Confirmatory factor analysis demonstrated good fit of this two-factor model. The internal consistency reliability, as measured by Cronbach’s α, was 0.85. The test-retest reliability coefficient was 0.7. The SCPSS-10 exhibited high correlation with the PHQ-9 and GAD-7, indicating an acceptable concurrent validity. The SCPSS-10 exhibited satisfactory psychometric properties in Chinese university students.

## Introduction

Psychological stress arises from an imbalance between individual’s perception and the external environmental demands. Studies have demonstrated that psychological stress is closely correlated with anxiety, depression, and physical conditions such as cardiovascular diseases and cancer [1–4]. Psychological stress reflects the subjective evaluation of one’s ability to cope with demands. People experience stress when they perceive that their resources are insufficient to cope with a situation [5].

University students encounter many sources of stress such as variable environment, lifestyle changes, academic burdens, and interpersonal relationships, all of which can lead to significant psychological dysfunctions. In particular, most university students in China are sensitive to stress since they tend to be the only child in their family. Indeed, studies have shown high levels of stress and depression in Chinese university students [6, 7].

The Perceived Stress Scale (PSS), one of the most widely used psychological scales, was developed by Cohen in 1983 and it has shown sufficient reliability and validity [8, 9]. The PSS has a number of advantages over other tools: 1) it only takes a few minutes and is easy to score; 2) the items are easily understandable; 3) it is not limited to a specific situation and can be used for past or ongoing events; 4) it can be used to examine changes over time in response to stress-inducing events; and 5) the PSS can be used as an outcome variable [9–11]. The PSS has been translated into many languages including Portuguese, Japanese, Arabic, Thai, and Chinese, as well as across several populations including pregnant and postpartum women, cardiac patients, and medical students [12–17]. The PSS was translated in Traditional Chinese, tested in Taiwan and Hong Kong, and found to be reliable [14, 18]. Nevertheless, there has only been one study on the validity of 10-item PSS in simplified Chinese (SCPSS-10), the primary language used in mainland China; this study was conducted in Chinese policewomen [17]. Thus, we analyzed the generalizability of the SCPSS-10 to other Chinese speaking populations.

Therefore, we assessed the perceived stress levels to evaluate the mental health status of Chinese university students. Perceived stress has not been measured and validated in this population. Thus, this study is the first to evaluate the reliability and validity of the SCPSS-10 in Chinese university students.

## Methods

### Subjects

Undergraduate students were recruited from Shanghai Jiao Tong University between January 1, 2011 and December 31, 2011. A total of 10 departments were selected from 22 faculties based on convenience sampling; 2191 students from the 10 departments were invited to participate in the study, of whom 1096 (50.0%) agreed to participate and completed the scales. Out of those students, 129 students (11.8%) were randomly selected for reassessment 2 weeks later using the SCPSS-10.

All the procedures were reviewed and approved by the Institutional Review Board of Shanghai Mental Health Center. Written informed consent was obtained from every subject before participation.

### Instruments

All the participants were asked to complete the following self-administered questionnaires in a classroom within 30 minutes. No more than 50 students completed the test simultaneously in the same classroom.

### Perceived Stress Scale-10

The Perceived Stress Scale (PSS) is a self-reported scale with three versions: 14-item scale, 10-item scale, and four-item scale. The 10-item version (PSS-10) has demonstrated good reliability and validity with a Cronbach’s α of 0.78–0.91 and test-retest reliability coefficients of 0.55–0.80 [8, 9, 19], i.e. with better reliability and validity than the other two versions [8]. The SCPSS-10, the Chinese version of the PSS-10, has shown good reliability and validity with a Cronbach’s α of 0.86 [14]. The version used in this study is the same as the one used in policewomen [14] and consists of all 10 original PSS items, six of which are negative (items 1, 2, 3, 6, 9, and 10) while the others are positive (items 4, 5, 7, and 8). The participants were required to answer each question using a five-point Likert scale score ranging from 0 (never) to 4 (very often) and report the event frequency correlated with the PSS items in the last month. Total scores ranged from 0 to 40, and participants with higher scores had higher perceived stress levels.

### Patient Health Questionnaire (PHQ-9)

The PHQ-9 is a self-reported version of the Primary Care Evaluation of Mental Disorders (PRIME-MD) diagnostic instrument for mental disorders in primary care settings, but not in psychiatric settings [20]. The PHQ-9 covers nine dimensions listed in the *Diagnostic and Statistical Manual of Mental Disorders*, 4^th^ Edition, and consists of nine items for measuring the frequency of depressive symptoms within 2 weeks [21, 22]. Each item ranges from 0 (not at all) to 3 (nearly every day). Total score represents the severity of depressive symptoms ranging from 0 to 27, and a score of 27 represents the most severe symptoms. Scores of 5, 10, 15, and 20 represent the threshold for mild, moderate, moderately severe, and severe depression. Previous studies demonstrated that the PHQ-9 is a reliable and robust instrument for screening depressive symptoms in adults [22, 23]. Studies have shown that perceived stress can result in the development of depression, and that the PSS-10 has a good reliability for estimating the level of stress perception [13]. The PHQ-9 has been translated into various languages (including Chinese) and yields robust reliability, with Cronbach’s α values of 0.73–0.95 [24–28].

### Generalized Anxiety Disorder 7-item scale (GAD-7)

The GAD-7 questionnaire [21] is a one-dimensional self-reported scale designed to assess the symptoms of anxiety. The GAD-7 has good reliability as well as criterion, construct, factorial, and procedural validity [21, 26, 29–31]. It consists of seven items for detecting the frequency of anxiety symptoms during the previous two weeks. Each item ranges from 0 (not at all) to 3 (nearly every day). The total scores range 0–21, and scores of 5, 10, and 15 represent the thresholds for mild, moderate, and serious depressive symptoms [21, 22, 24–28]. Although anxiety is a normal reaction to stress, in excessive or chronic cases anxiety disorders may develop. Previous studies found a high correlation between anxiety measures and the PSS-10, indicating concurrent validity of the scales [32]. The Chinese version of the GAD-7 has demonstrated good psychometric properties [33].

### Statistical analysis

Continuous variables were presented as mean and standard deviation. The internal consistency reliability of the SCPSS-10 was evaluated using the Cronbach’s α coefficient. A Cronbach’s α coefficient value of 0.7–0.8 indicated sufficient reliability, whereas a value of 0.8–0.9 indicated very good reliability. Pearson correlation analysis was applied to assess the test-retest reliability and evaluate the concurrent validity of SCPSS-10 on the depressive and anxious symptoms measured by the PHQ-9 and GAD-7.

The samples were randomly split into two halves for the construct structure analysis. The sample data were screened to confirm that no assumptions were violated prior to the EFA using the Kaiser-Meyer-Olkin test and Bartlett’s test of sphericity. Exploratory factor analysis (EFA) was conducted with the first half using the principle component analysis with varimax rotation. The Kaiser-Meyer-Olkin measure of sampling adequacy was applied to assess sample adequacy. Confirmatory factor analysis (CFA) was applied to determine the fitness of the previously identified two-factor and one-factor models [19]. The covariance matrix was tested by the maximum-likelihood estimation method to determine how well the model fitted the sample data. Goodness-of-fit index (GFI), normalized fit index (NFI), comparative fit index (CFI), root mean square residual (RMSR), and root mean square error of approximation (RMSEA) were used to evaluate the models.

SPSS version 20.0 (IBM, Armonk, NY, USA) was used for analysis. The CFA analysis was conducted using AMOS 7.0 (SPSS Inc., Chicago, IL, USA). *p* < 0.05 was considered statistically significant.

## Results

### Participant characteristics

The mean age of the 1096 participants (395 women, 701 men) was 18.3 ± 0.7 years. The mean SCPSS-10 score was 13.7 ± 5.6 for the whole student sample, and there were no significant differences between females (13.9 ± 5.5) and males (13.5 ± 5.5).

For the whole study sample, the SCPSS-10, PHQ, and GAD scores were 13.7±5.6, 4.3±3.1, and 2.8±2.9, respectively. For the retest sample, the SCPSS-10, PHQ, and GAD scores were 18.6±4.7, 4.9±3.2, and 3.6±3.3, respectively.

### Reliability of the SCPSS-10

The internal consistency of the SCPSS-10 was reliable (Cronbach’s α = 0.85) for the whole sample set. At the end of the second week, the test-retest reliability of the SCPSS-10 was 0.70.

### EFA

Sampling adequacy was good (Kaiser-Meyer-Olkin = 0.86) and Bartlett’s test of sphericity was statistically significant (*p* < 0.001). The principal component analysis with the varimax rotation of the SCPSS-10 scores obtained two factors with Eigen values > 1.0 accounting for 62.49% of the variance (Table 1). Factor loading ranged from 0.67 to 0.80 for SCPSS items, which entered into factor 1, and from 0.73 to 0.87 for SCPSS items, which entered into factor 2.

**Table 1:** Exploratory factor analysis and reliability coefficients of the SCPSS-10 (n = 548).

SCPSS-10 Item	Factor loading	
	Factor 1	Factor 2
1. In the last month, how often have you been upset because of something that happened unexpectedly?	0.777	0.040
2. In the last month, how often have you been unable to control the important things in your life?	0.755	0.188
3. In the last month, how often have you felt nervous and “stressed”?	0.793	0.070
4. In the last month, how often have you felt confident about your ability to handle your personal problems?	0.146	0.844
5. In the last month, how often have you felt that things were going your way?	0.349	0.732
6. In the last month, how often have you found that you could not cope with all of the things that you had to do?	0.723	0.211
7. In the last month, how often have you been able to control irritations in your life?	0.014	0.738
8. In the last month, how often have you felt that you were in control of things?	0.214	0.869
9. In the last month, how often have you been angered because of things that were outside of your control?	0.667	0.165
10. In the last month, how often have you felt difficulties were piling up so high that you could not overcome them?	0.756	0.237
Eigen value	4.43	1.81
% variance	44.34	18.14
Cronbach’s α coefficient	0.86	0.83

SCPSS-10, Simplified Chinese version of the 10-item Perceived Stress Scale.

### CFA

In this sample, the two-factor solution was demonstrated to be adequate: GFI = 0.940, NFI = 0.925, CFI = 0.939, RMSR = 0.039, and RMSEA = 0.049. In contrast, the one-factor model showed a poor data fit: GFI = 0.676, NFI = 0.596, CFI = 0.628, RMR = 0.093, and RMSEA = 0.122. None of the fit indices matched the cut-off criterion (Table 2). The CFA indicated that the SCPSS-10 two-factor model was a reasonable approximation to the population.

**Table 2:** Goodness-of-fit indices of two CFA models of the SCPSS-10 (n = 548).

Model	GFI	NFI	CFI	RMR	RMSEA
One-factor model	0.676	0.596	0.628	0.093	0.122
Two-factor model	0.940	0.925	0.939	0.039	0.049

### Concurrent validity

We analyzed the association between the SCPSS-10, GAD-7, and PHQ-9 (Table 3). GAD-7 and PHQ-9 were positively correlated with SCPSS-10. The factors associated with the SCPSS-10 total score and the other two scales had correlation coefficients of 0.37–0.87 (Table 3).

**Table 3:** Correlations between SCPSS-10, depression (PHQ-9), and anxiety (GAD-7) screening tools.

	SCPSS-10	Factor 1	Factor 2	GAD-7
Factor 1	0.87			
Factor 2	0.77	0.37		
GAD-7	0.59	0.56	0.39	
PHQ-9	0.57	0.53	0.40	0.72

SCPSS-10, Simplified Chinese version of the 10-item Perceived Stress Scale; PHQ-9, Patient Health Questionnaire; GAD-7, Generalized Anxiety Disorder 7-item scale.

## Discussion

Our study assessed for the first time perceived stress in Chinese university students. The psychometric data yielded good validity and reliability for SCPSS-10. Overall, the Cronbach’s α of the SCPSS-10 was 0.85, which is better than the standard of psychological measurement (Cronbach’s α > 0.7) [34]. In addition, the 2-week test-retest reliability of the SCPSS-10 was 0.70, which is acceptable and demonstrated good cross-temporal constancy, similar to our previously published studies on policewomen (*r* = 0.68). These results are consistent with previous studies of the PSS in other languages [2, 13–16, 19] as well as previous study from our group conducted in Chinese policewomen, in which we obtained a Cronbach’s α value of 0.86 [17]. SCPSS-10 revealed high levels of perceived stress in Chinese university students, which also correlated with anxiety and depression.

Regarding the SCPSS-10 structure, the construct validity analysis showed that the two common factors extracted from the principal component analysis with varimax rotation represented positive (Factor 1) and negative (Factor 2) feelings. This was consistent with other language versions [8, 12, 13]. In this study, the Eigen values of the SCPSS-10 were 4.43 and 1.81, accounting for 44.34% and 18.14% of the variance. The loadings were 0.667–0.793 for Factor 1 and 0.732–0.869 for Factor 2. The CFA demonstrated a relatively better goodness-of-fit for the two-factor model for SCPSS-10 compared with the previous studies [17]. This may be due to higher homogeneity of the sample, as undergraduate students tend to have similar economic, social, and cultural backgrounds compared to policewomen. Although some researchers have suggested one-factor model solution for the PSS-10, CFA revealed a poor fit using this model. These results were consistent with a previous report based on Chinese speaking population [35]. Furthermore, the correlation coefficients between the two extracted common factors and the total SCPSS-10 scores were 0.87 and 0.77, and the correlation coefficient between Factors 1 and 2 was 0.37, indicating good internal homogeneity. Nevertheless, “positive feelings” and “negative feelings” both reflect perceived stress, and it has been suggested that any distinction between these factors is irrelevant and reflects the sentence structure of the scale [8]. Thus, although the two-factor model solution of PSS-10 is better, we suggest not using two separate subscales in the clinical setting.

Wang et al. demonstrated that the SCPSS-10 was significantly and moderately positively correlated with anxiety and depression in policewomen [17]. Other studies have showed that the PSS-10 has concurrent validity with other measures, including the State Trait Anxiety Inventory and the Beck Depression Inventory [13, 36]. We found that SCPSS-10 is moderately positively correlated with PHQ-9 (r_s_ = 0.59, *p* < 0.001) and GAD-7 (r_s_ = 0.57, *p* < 0.001).

For the satisfactory psychometric results in Chinese university students, we propose that SCPSS-10 should be widely applied in Chinese speaking university students, even outside China mainland. Nevertheless, because of differences in university levels, and of social, economic, and cultural differences among Chinese-speaking regions, additional studies should be performed in those regions in order to confirm the generalizability of our results. Although the participants recruited in our study were all freshmen university students, those in higher grades may have different levels and kinds of stress, which may require different evaluation. For example, junior, senior, or graduate-level students may encounter higher psychological stress because of the accumulated study burden and job-hunting pressures. University students may also have different perceived psychological pressures depending on their educational levels, although both groups in the present study received similar education at similar ages after high school. Second, construct validity was limited to self-reported measure comparisons. Third, we did not evaluate discriminant validity of the SCPSS-10 in this present study. Thus, further studies should address these questions in diverse populations, and more objective measures should be applied to improve the psychometric quality of the SCPSS-10.

## Conclusion

In conclusion, we find that SCPSS-10 is a reliable and valid instrument for estimating the stress levels in university students within a Chinese cultural context. We also found high perceived stress levels in university students, which was also correlated with anxiety and depression.

## Supporting information

S1 Table Stress scale retest list and results. (XLSX)

S2 Table Stress test list and results. (XLSX)
